# Supplementary material for: Strainberry: automated strain separation in low-complexity metagenomes using long reads
Source: Nat Commun. 2021 Jul 23;12:4485. doi: 10.1038/s41467-021-24515-9 (PMC8302730; doi:10.1038/s41467-021-24515-9)
Supplement: Supplementary file 1 — Supplementary Information [file 41467_2021_24515_MOESM1_ESM.pdf]

Supplementary Information

Strainberry: automated strain separation in low-complexity metagenomes using long reads

Riccardo Vicedomini[1]*, Christopher Quince[2,3,4], Aaron E. Darling[5], Rayan Chikhi[1]

[1] Sequence Bioinformatics, Department of Computational Biology, Institut Pasteur, Paris, France
[2] Organisms and Ecosystems, Earlham Institute, Norwich, United Kingdom
[3] Gut Microbes and Health, Quadram Institute, Norwich, United Kingdom
[4] Warwick Medical School, University of Warwick, Coventry, United Kingdom
[5] The iThree Institute, University of Technology Sydney, Ultimo, Australia

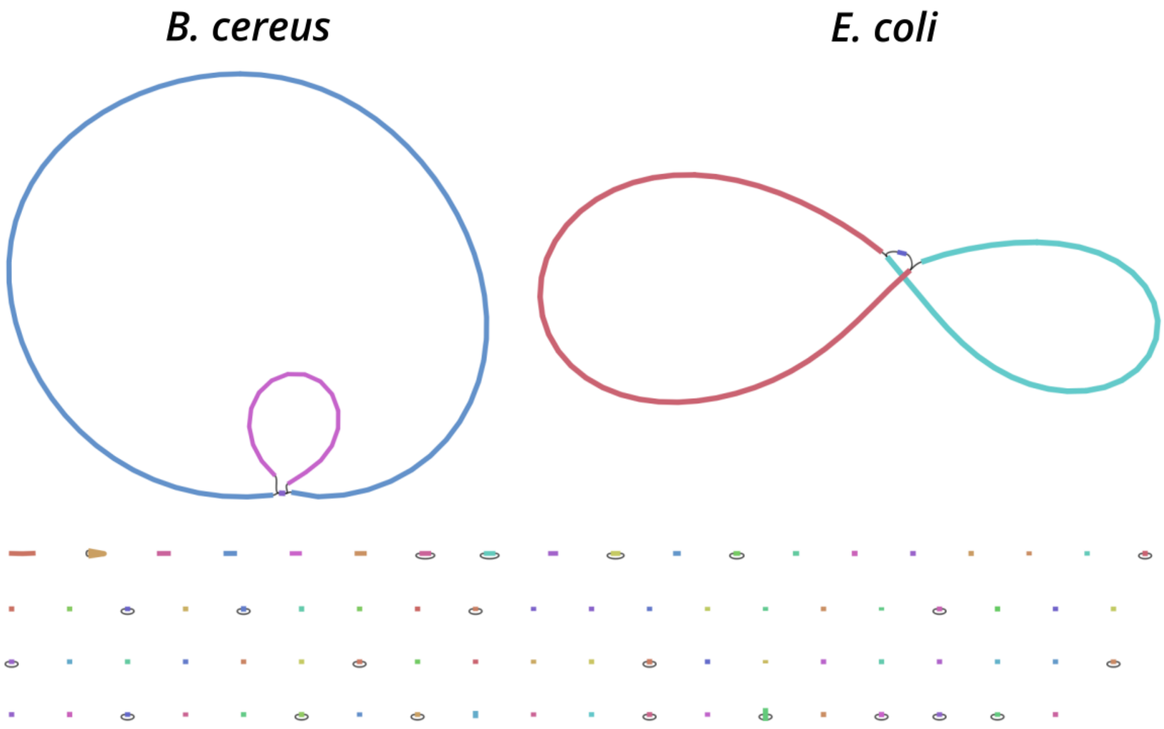

Supplementary Figure 1. Bandage plot of the Flye assembly graph of the Mock3 dataset. The picture shows assembled sequences of metaFlye for the Mock3 dataset. Lines correspond to contigs. Edges, in the form of thin black lines between the contigs, reflect adjacencies. Colors are arbitrary. While *B. cereus* genome was correctly reconstructed, *E. coli* strains K-12 and W have been mainly collapsed into two long consensus sequences. At the same time many "short" fragments appear due to genomic sequences specific to either one of the two strains.

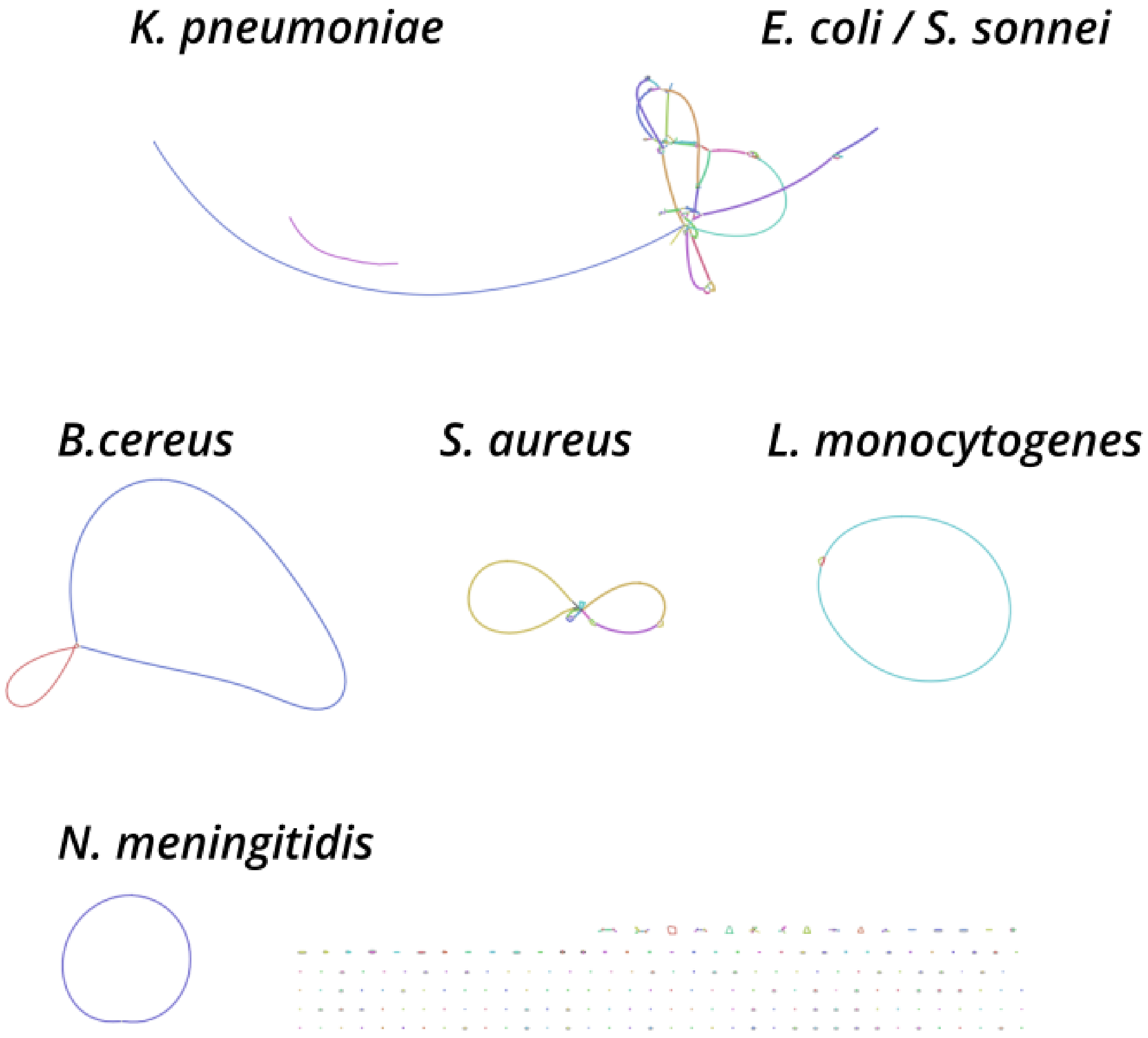

Supplementary Figure 2. Bandage plot of Flye assembly graph of the Mock9 dataset. The picture shows assembled sequences of metaFlye for the Mock9 dataset (see caption of Supplementary Figure 1 for additional explanations). While single-strain genomes have been correctly reconstructed, the two *S. aureus* strains are mostly collapsed into long consensus sequences. Moreover, the assembly graph exhibits a more complex structure for the two *E. coli* strains due to the high similarity of the *S. sonnei* genome. As in Mock3, “short” fragments appear due to strain-specific sequences.

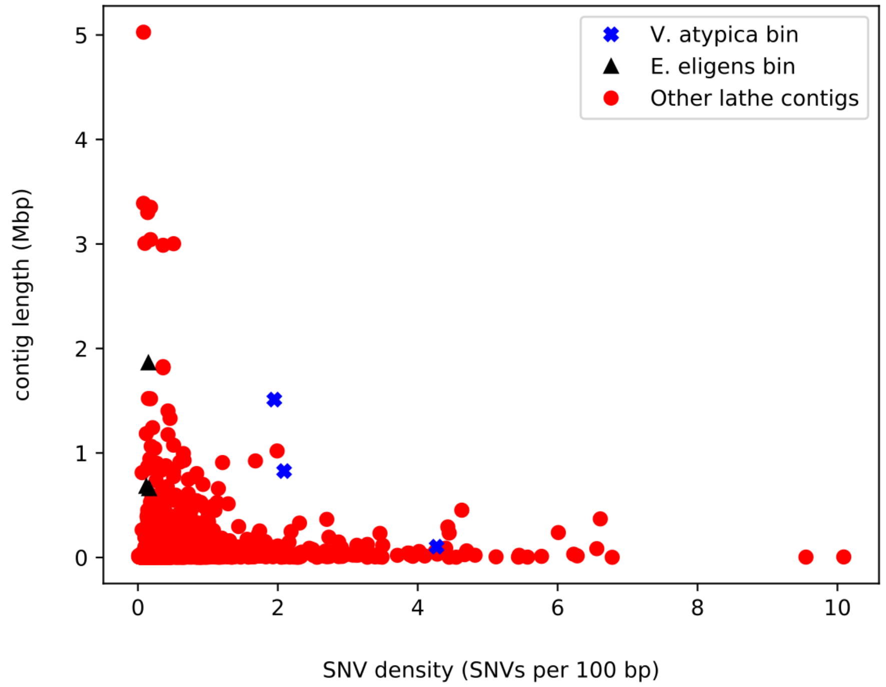

Supplementary Figure 3. SNV density of Lathe assembled contigs. Each red circle corresponds to a contig of the Lathe reference metagenome. Contigs of *Veillonella atypica* and *[Eubacterium] eligens* bins are emphasized with blue crosses and black triangles, respectively.

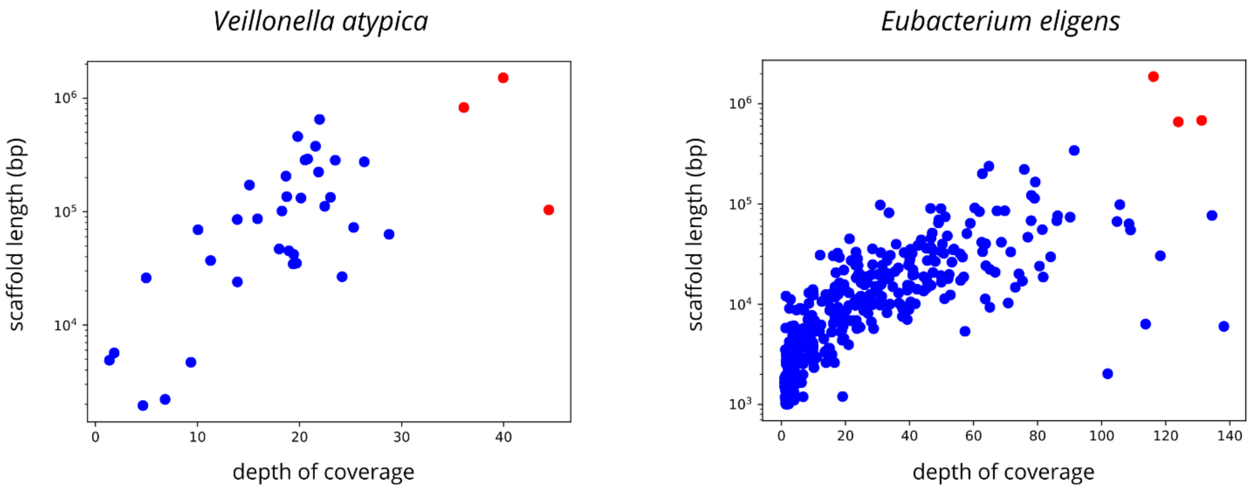

Supplementary Figure 4. Depth of coverage of *V. atypica* and *E. eligens* Lathe bins before and after the strain separation of the Lathe reference assembly. Red circles correspond to Lathe contigs. Blue circles correspond to Strainberry scaffolds. The y-axis is represented with a logarithmic scale.

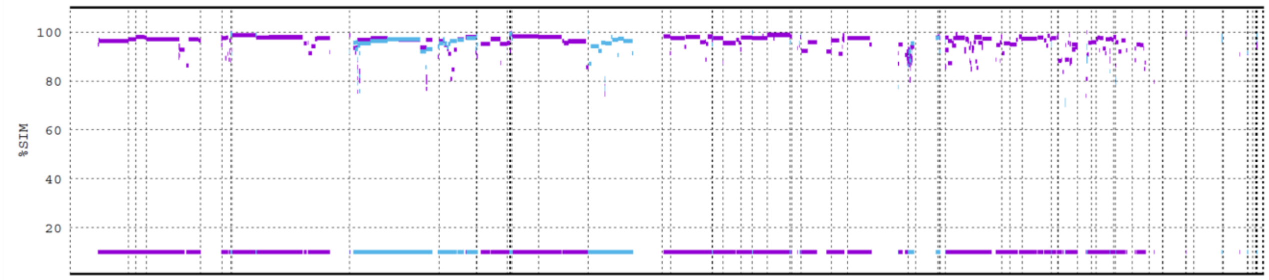

Veillonella atypica ACS-134-V-Col7a

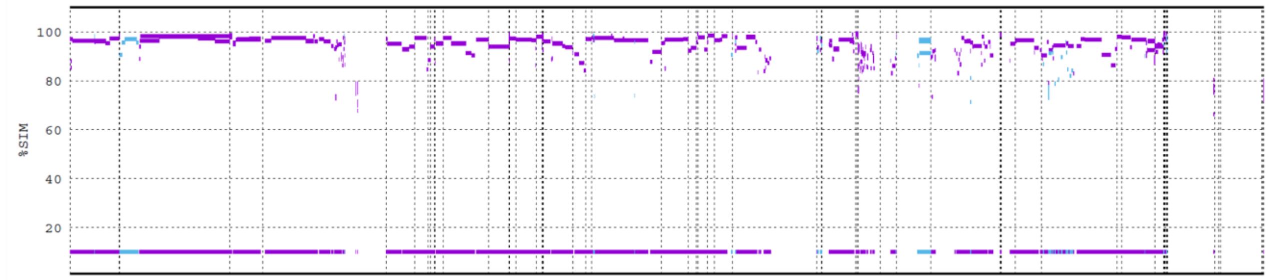

Veillonella atypica ACS-049-V-Sch6

Supplementary Figure 5. Mapping of the strain-separated contigs of the *V. atypica* bin classified as *V. atypica* strain ACS-134-V-Col7a and *V. atypica* strain ACS-049-V-Sch6 on the available NCBI contig-level references. The x-axis and y-axis represent the reference contigs and the sequence identity of mapped segments, respectively. The coverage of mapped segments onto the references is represented by a projection at $y = 10\%$. Overall, both references were covered at around 70%.

Supplementary Table 1. Mock datasets composition. Strains, genome size, reads, and coverage of the mock communities created from the PacBio Sequel data available at https://github.com/PacificBiosciences/DevNet/wiki/Microbial-Multiplexing:-PacBio-Sequel-System,-Chemistry---v3.0,-Analysis---SMRT-Link-v6.0.0. Reads sequenced with barcode BC1022 are supposed to come from *Staphylococcus aureus* strain HPV107, however through a BLAST alignment of the available isolate PacBio assembly against the RefSeq genome database we observed they likely come from *Staphylococcus aureus* strain FDAARGOS 766.

mock dataset	species	strain	RefSeq	genome size (bp)	barcode	reads	depth of coverage
Mock3	*Bacillus cereus*	ATCC 14579	NZ_CP034551.1	5,416,249	BC1010	402,216	447.13
	Escherichia coli	K-12 substr MG1655	NC_000913.3	4,641,652	BC1002	221,080	311.24
	Escherichia coli	W ATCC 9637	NC_017664.1	4,897,452	BC1019	360,041	435.21
Mock9	*Bacillus cereus*	ATCC 14579	NZ_CP034551.1	5,416,249	BC1010	402,216	447.13
	Escherichia coli	K-12 substr MG1655	NC_000913.3	4,641,652	BC1001	249,861	367.11
					BC1002	221,080	311.24
	Escherichia coli	W ATCC 9637	NC_017664.1	4,897,452	BC1019	360,041	435.21
	Klebsiella pneumoniae	ATCC BAA-2146	NZ_CP006659.2	5,435,746	BC1009	222,032	280.03
	Listeria monocytogenes	CFSAN008100	NZ_CP011398.2	3,039,878	BC1012	180,478	411.58
	Neisseria meningitidis	FAM18	NC_008767.1	2,194,961	BC1016	188,874	474.75
	Staphylococcus aureus	ATCC 25923	NZ_CP009361.1	2,778,854	BC1018	302,192	686.15
	Staphylococcus aureus	FDAARGOS 766	NZ_CP041010.1	2,877,716	BC1022	221,177	543.55
	Shigella sonnei	CFSAN030807	NZ_CP023645.1	4,813,450	BC1015	237,599	333.39

Supplementary Table 2. Reference sequences of simulated datasets. RefSeq accession codes of the reference genomes used to generate the simulated datasets with variable strain divergence, number of strains, and recombination rate.

simulated mock community	species	strain	RefSeq	genome size (bp)
variable strain divergence	*Escherichia coli*	K-12 substr MG1655	NC_000913.3	4,641,652
		ME8067	NZ_CP028703.1	4,614,635
		LD27-1	NZ_CP047594.1	4,694,065
		Y5	NZ_CP013483.1	4,839,266
		EC590	NZ_CP016182.2	4,617,703
		RM14721	NZ_CP027105.1	4,726,450
		AMSCJX03	NZ_CP058355.1	4,819,811
		H5	NZ_CP010169.1	4,833,228
variable number of strains	*Escherichia coli*	K-12 substr MG1655	NC_000913.3	4,641,652
		W ATCC 9637	NC_017664.1	4,897,452
		H5	NZ_CP010169.1	4,833,228
		LD39-1	NZ_CP047658.1	4,684,883
		AMSCJX03	NZ_CP058355.1	4,819,811
variable recombination rate	*Buchnera aphidicola*	Bp (Baizongia pistaciae)	NC_004545.1	615,980
		W106 (Myzus persicae)	NZ_CP002699.1	643,502
	Escherichia coli	K-12 substr MG1655	NC_000913.3	4,641,652
		W ATCC 9637	NC_017664.1	4,897,452
	Helicobacter pylori	ASHA-004	NZ_CP051434.1	1,615,144
		ASHA-005	NZ_CP051435.1	1,630,559
	Neisseria meningitidis	09-292	NZ_CP021521.1	2,198,497
		11-7	NZ_CP021520.1	2,157,444

Supplementary Table 3. Assembly evaluation of the Strainberry separated assemblies of the NWC2 Nanopore dataset, before and after polishing. The following quantitative and qualitative measures are displayed for each assembly: assembly method, reference sequence, number of contigs (#seq), reference size (ref size), assembly size (asm size), NG50, unaligned reference percentage (unaligned ref %), unaligned assembly percentage (unaligned asm %), average nucleotide identity (ANI), duplication ratio (dup ratio), duplicated bases (dup), compressed bases (cmp), single-nucleotide polymorphisms (SNPs), inversions (inv), relocations (reloc). Completeness (compl), contamination (contam), and strain heterogeneity (s.h.) refer to the metrics computed with CheckM on assembled sequences assigned to each reference.

assembly	reference	#seq	ref size (bp)	asm size (bp)	NG50	unaligned ref %	unaligned asm %	ANI	dup ratio	dup	cmp	SNPs	inv	reloc	compl	contam	s.h.
**Flye**	*S. thermophilus* NWC_2_1	8	1,971,439	2,010,238	1,929,846	0.00	0.29	99.70	1.02	38,752	0	1,033	0	1	79.9	0.2	0.0
	L. delbrueckii NWC_2_2	60	2,269,179	3,210,504	136,620	0.17	3.46	99.57	1.37	949,027	64,371	2,555	2	6	92.2	29.6	100.0
	L. helveticus strain **NWC_2_3**	**106**	**2,210,811**	**1,831,152**	**49,497**	**34.32**	**3.68**	**99.41**	**1.21**	**552,253**	**296,670**	**3,386**	**9**	**8**	44.5	6.9	100.0
	L. helveticus strain **NWC_2_4**	**78**	**2,177,422**	**2,725,531**	**190,476**	**6.69**	**1.12**	**99.76**	**1.33**	**708,023**	**68,080**	**541**	**4**	**5**	89.2	3.5	100.0
**ssFlye**	*S. thermophilus* NWC_2_1	8	1,971,439	2,010,238	1,929,846	0.00	0.29	99.70	1.02	38,752	0	1,033	0	1	79.9	0.2	0.0
	L. delbrueckii NWC_2_2	112	2,269,179	4,818,600	173,853	0.36	2.44	98.78	2.08	2,596,568	85,545	3,907	2	1	80.2	58.9	58.3
	L. helveticus strain **NWC_2_3**	**139**	**2,210,811**	**3,000,934**	**83,907**	**9.34**	**1.79**	**99.38**	**1.47**	**1,017,479**	**104,941**	**1,798**	**1**	**2**	64.2	14.5	50.0
	L. helveticus strain **NWC_2_4**	**107**	**2,177,422**	**3,310,089**	**144,095**	**5.03**	**2.01**	**99.49**	**1.57**	**1,205,697**	**53,972**	**620**	**1**	**2**	83.3	37.4	70.5
**ssFlye + Medaka**	*S. thermophilus* strain NWC_2_1	4	1,971,439	1,981,750	1,922,746	0.18	0.29	99.49	1.00	18,016	15,546	562	0	11	82.7	0.9	0.0
	L. delbrueckii strain NWC_2_2	64	2,269,179	4,656,990	175,783	0.67	2.42	99.16	2.02	2,362,197	75,047	3,221	1	4	88.6	73.6	81.5
	L. helveticus **strain NWC_2_3**	**67**	**2,210,811**	**2,771,930**	**82,042**	**6.16**	**1.60**	**99.39**	**1.31**	**669,397**	**89,603**	**1,854**	**1**	**3**	80.9	16.2	73.1
	L. helveticus **strain NWC_2_4**	**76**	**2,177,422**	**3,060,045**	**125,213**	**3.92**	**1.59**	**99.46**	**1.44**	**900,321**	**54,029**	**767**	**1**	**1**	88.8	22.2	78.6
**ssFlye + MarginPolish**	*S. thermophilus* strain NWC_2_1	4	1,971,439	1,997,139	1,937,937	0.18	0.30	99.46	1.01	19,586	0	1,122	0	1	84.2	0.0	0.0
	L. delbrueckii strain NWC_2_2	59	2,269,179	4,524,070	175,547	2.70	3.88	98.74	1.97	2,211,349	65,296	5,739	4	2	72.9	28.9	52.8
	L. helveticus **strain NWC_2_3**	**53**	**2,210,811**	**2,418,007**	**81,656**	**10.86**	**2.50**	**99.28**	**1.20**	**481,168**	**146,765**	**1,996**	**1**	**2**	64.2	16.3	46.7
	L. helveticus **strain NWC_2_4**	**61**	**2,177,422**	**2,992,225**	**145,301**	**1.83**	**4.65**	**99.22**	**1.33**	**712,862**	**45,756**	**2,661**	**2**	**2**	89.2	35.1	86.7

Supplementary Table 4. CheckM evaluation of the *V. atypica* and *E. eligens* bins of the HSM dataset. Completeness, contamination and strain heterogeneity (s.h.) percentages of the Lathe reference assembly compared to the Strainberry-separated scaffolds from the Lathe assembly (ssLathe) and the Flye assembly. Flye sequences were selected as the best-mapping ones against Lathe sequences.

assembly	species	size (Mbp)	completeness	contamination	s.h.
**Lathe**	*V. atypica*	2.44	93.9	1.5	100.0
	E. eligens	3.20	87.6	0.0	0.0
**ssLathe**	*V. atypica*	4.50	67.3	33.2	63.6
	E. eligens	7.66	48.6	16.4	49.3
**ssLathe/polished**	*V. atypica*	4.55	84.5	71.5	73.4
	E. eligens	7.20	89.0	74.2	81.7
**Flye**	*V. atypica*	2.19	57.0	2.4	81.8
	E. eligens	2.64	69.2	0.0	0.0
**Flye/polished**	*V. atypica*	2.35	64.1	3.1	28.6
	E. eligens	2.63	70.9	0.3	0.0

Supplementary Table 5. Runtime and memory usage of Strainberry on the different datasets. Time and memory needed to generate the input assembly (Flye), perform the read mapping (Minimap2), and separate the assembly with Strainberry. The estimated reference size of the mock and HSM datasets refers to the size of available references, while for NWC2 it refers to the size of the Flye assembly.

dataset	size (Gbp)	mean coverage	est. ref. size (Mbp)	tool	wall-clock time (12 threads)	CPU time	RAM peak (GB)
**Mock3**	6.00	515X	14.96	*Flye*	2h 52m 46s	18h 54m 25s	20.36
	6.00	515X		*Minimap2*	6m 39s	1h 5m 49s	9.31
	0.90	75X		*Strainberry*	31m 48s	2h 55m 1s	4.56
**Mock9**	16.59	460X	36.18	*Flye*	7h 8m 18s	56h 19m 30s	132.80
	16.59	460X		*Minimap2*	20m 52s	3h 28m 13s	9.37
	2.48	86X		*Strainberry*	1h 48m 12s	12h 27m 42s	6.17
**NWC2-PacBio**	4.21	372X	9.98	*Flye*	6h 29m 38s	50h 45m 25s	25.12
	4.21	372X		*Minimap2*	9m 26s	1h 45m 18s	9.26
	0.73	66X		*Strainberry*	13m 28s	2h 0m 7s	7.19
**NWC2-ONT**	0.67	63X	10.10	*Flye*	1h 1m 55s	7h 56m 44s	35.96
				Minimap2	1m 58s	14m 32s	4.56
				Strainberry	17m 21s	2h 25m 58s	3.89
**HSM**	11.36	61X	162.81	*Flye*	6h 34m 2s	54h 51m 22s	131.97
				Minimap2	18m 12s	3h 6m 38s	9.46
				Strainberry	3h 59m 19s	41h 30m 52s	9.46

Supplementary Table 6. Runtime and memory usage of the pipeline using different combinations of SNV-detection and haplotype-phasing tools on the Mock3 dataset.

method (12 CPUs)	haplotype separation	haplotype assembly	scaffolding	total	RAM peak (GB)
**Longshot +HapCUT2**	17m 36s	12m 32s	1m 40s	31m 48s	4.56
**Longshot +whatshap-polyploid**	41m 35s	15m 0s	2m 1s	58m 36s	4.04
**freebayes +whatshap-polyploid**	26h 41m 44s	12m 37s	1m 57s	26h 56m 18s	4.09

Supplementary Note 1: Analysis of Mock3 assemblies

Note: in all our tests on the Mock3 dataset we used a beta version of Flye (v2.7b-b1528), which turns out to produce modestly better assemblies than the latest Flye release at the time of experiments (v2.7-b1585).

For the genome with only one strain present (*B. Cereus*) the NG50 of Strainberry+Flye scaffolds (5.4 Mbp) is higher than Flye (4.3 Mbp), due to additional scaffolding. For *E. coli* strain K12, Flye reports a NG50 of zero due to the low number of assembled bases, while the strain-separated assembly achieves a NG50 of 1.28 Mbp. For *E. coli* strain W, the strain-separated scaffolds have lower NG50 (1.34 Mbp) than Flye (2.83 Mbp) since we report haplotype blocks while Flye contigs correspond to a strain consensus. There is therefore a trade-off between high NG50 and strain reconstruction accuracy.

As misassemblies are concerned, the Strainberry+Flye scaffolds yielded 2 relocations for B. cereus which were also present in the upstream Flye assembly (both due to the circularity of the genome and not actual rearrangements). The 3 new relocations in the Strainberry+Flye assembly of *E. coli* strain K12 are due to the circularity of the genome and the introduction of small gaps (123 bp and 211 bp) during the scaffolding phase. With respect to the *E. coli* strain W reference, Strainberry+Flye is affected by the same number of misassemblies of the Flye assembly. More precisely, one relocation is due to the circularity of the genome for both the assemblies, while the other two to unresolved repeats (2 Kbp and 5 Kbp) for Strainberry and two small insertions in the assembly for Flye (25 bp and 922 bp). Finally, inversions are identified on non-separated contigs and therefore are inherited by the Flye assembly.

Supplementary Note 2: Analysis of Mock9 assemblies

For the genome with only one strain present (*B. cereus*, *K. pneumoniae*, *L. monocytogenes*, *N. meningitidis*) the strain-oblivious assemblies achieved a near-complete genome reconstruction with average nucleotide identity greater than 99.9% and an optimal duplication ratio. Strainberry scaffolds yielded comparable results as no major separation was performed. For *S. aureus* strain FDAA, Flye reports a NG50 of 1.52 Mbp, while the strain-separated assembly achieves a NG50 of 2.67 Mbp. For *S. aureus* strain ATCC, the strain-separated scaffolds yield 2.80 Mbp of assembled bases (with a NG50 of 2.20 Mbp) compared to the 719 Kbp of assembled bases yielded by Flye. Canu yielded an NG50 of 2.44 Mbp and 56 Kbp for strains FDAA and ATCC, respectively. Even though Strainberry was able to achieve a complete coverage of the two *S. aureus* strains, the high duplication ratio of Canu assembly hindered the scaffolding of the strain-separated contigs leading to a NG50 of 82 Kbp and 60 Kbp for strains FDAA and ATCC, respectively. For *E. coli* strains K12, W and *S. sonnei*, Flye reports a NG50 of 3 Kbp and 60 Kbp for *E. coli* strain K-12 and *S. sonnei*, respectively, but it was not able to cover half of the reference of *E. coli* strain W. While significantly improving reference coverage and sequence identity, the three-strain separation achieves a NG50 of 58 Kbp, 44 Kbp, and 62 Kbp, for *E. coli* strains K12, W and *S. sonnei* respectively, highlighting the difficulty of separating more than two strains into a contiguous set of scaffolds. On the other hand, Canu reports a NG50 of 543 Kbp, 41 Kbp and 44 Kbp, for *E. coli* strains K12, W and *S. sonnei,* respectively. The two-strain separation of Strainberry achieves instead a NG50 of 46 Kbp, 30 Kbp, and 34 Kbp, respectively, due to the difficulty of scaffolding a set of contigs characterized by a high duplication ratio. Misassemblies are precisely reported in Supplementary Data 1.

Supplementary Note 3: Analysis of NWC2 PacBio assemblies

Compared to the Flye strain-oblivious assembly, the strain-separated assembly is of higher quality as evidenced by comparable and/or improved values of NG50, sequence identity, and misassemblies. Regarding the separation of the two *L. helveticus* strains, for instance, strain-separated scaffolds of NWC_2_4 improved NG50 (Strainberry attained 146 Kbp, while Flye yielded 92 Kbp.) Sequence identity of strain NWC_2_3 was also improved by Strainberry (99.74% versus 99.60% with Flye). For *L. delbrueckii*, strain-separated sequences achieve a higher NG50 (224 Kbp) compared to Flye (190 Kbp). At the same time, the higher assembly size and duplicated bases are likely due to the potential existence of other undetected conspecific strain(s) of *L. delbrueckii*, as also evidenced by the strain-separated assembly obtained with the longer Nanopore reads. For all the four reference strains, the number of inversions and relocations of separated contigs/scaffolds is either comparable to the Flye assembly or decreased due to a correct strain reconstruction. Compared to the Canu assembly, the strain-separated assembly reduced the number of detected misassembly events and improved the reference coverage of *L. helveticus* strains at the cost of a reduced NG50 (see Supplementary Data 2).

Supplementary Note 4: Analysis of NWC2 Nanopore assemblies

Results obtained with Nanopore sequencing data follow the same trend as with the PacBio data. A key difference lies in the number of misassembly events detected after Strainberry separation which reduced with respect to the Flye assembly but also compared to the strain-separated assembly obtained with PacBio data. The NG50 for *L. helveticus* strains also displays a different behavior for the Flye Nanopore-based assembly which doubled for strain NWC_2_4 but dropped by half for strain NWC_2_3. Strain-separated assemblies however show comparable values of NG50 between the two technologies. Moreover, Nanopore-based strain-separated assemblies have lower sequence identities to reference strains (98.78%-99.70%) than with PacBio data (99.74%-99.92%). Also, the lower sequence identities in the strain-aware assemblies, compared to the strain-oblivious assembly, are likely caused by splitting reads into two haplotype groups, thus having less input coverage. We attempted to mitigate this effect by polishing the strain-separated contigs using Nanopore-specific tools such as Medaka (https://nanoporetech.github.io/medaka) and MarginPolish (https://github.com/UCSC-nanopore-cgl/MarginPolish), with limited success (Supplementary Table 3). The assembly evaluation also shows a 1.6-Mbp increase of strain-resolved sequences aligning against the *L. delbrueckii* reference compared to the Flye assembly. This is further evidence that, thanks to the longer Nanopore reads, a conspecific strain might have been separated by Strainberry. As for the Canu assembly, compared to the PacBio data, results show again a similar trend, yet with larger values of NG50 and an increased number of misassembly events (Supplementary Data 2). The strain-separated assembly slightly improves reference coverage of *L. helveticus* strain NWC_2_3 and increases the assembly size of scaffolds aligning to *L. delbrueckii*. It yields however comparable results according to the other evaluation metrics. As in the mock datasets, Canu reports a very high number of duplicated sequences which, again, could be due to erroneous duplications or correctly separated conspecific strains. *L. delbrueckii* reference is largely covered two and three times by the Canu and Strainberry assembly respectively. This higher coverage is likely due to a conspecific strain (as observed for the strain-separated Flye assembly and with PacBio data). There is however the possibility that the separation performed by Canu is not optimal and could affect the performance of Strainberry, as seen for the Mock3 and Mock9 datasets.

As the CheckM analysis is concerned, compared to the Flye assembly, strain-separated sequences globally show higher completeness for *L. helveticus* strains (from 44.5% to 64.2% with NWC_2_3 strain-separated scaffolds). On the contrary, *L. delbrueckii* sequences are characterized by a slightly lower completeness (92.2% in strain-oblivious assembly, 80.2% in strain-aware) and a much higher contamination (29.6% in strain-oblivious assembly, 58.9% in strain-aware). On the other hand, the Canu assembly and the related strain-separated Strainberry assembly yield comparable metrics.

Supplementary Note 5: HSM dataset evaluation

Evaluation metrics of the assembled sequences were computed using QUAST v5.1.0rc1 with parameters `--fragmented --min-identity 80 --min-contig 200`.

The binning of the Lathe assembly was generated with MetaBAT v2.15. First, the average depth of coverage of each assembled contig was computed using the command `jgi_summarize_bam_contig_depths` with parameters `--minContigLength 1000 --minContigDepth 1 --percentIdentity 50`. The actual binning was then produced using the `metabat2` command with default parameters.

Bin and read classifications were performed using Kraken (version 2.0.9-beta) with the Maxikraken2 database (update of March 2019) retrieved at https://lomanlab.github.io/mockcommunity/mc_databases.html. In addition to sequence hashes from genomes of NCBI RefSeq that are marked as complete or representative, it includes genomes that are either incomplete or not representative for the following database types: archaea, bacteria, fungi, protozoa, viral, and human.

Due to the higher complexity of the dataset, Strainberry was run independently on each bin. In order to fairly compare Lathe bin classification before and after the strain separation, separated bins were polished as it is done in the Lathe workflow: four rounds of Racon and one round of Medaka (see https://github.com/bhattlab/lathe).

Plots showing contig length and mean coverage of the Lathe and strain-aware assemblies of *V. atypica* and *E. eligens* bins were generated using a custom Python script.

In order to identify the Flye contigs that corresponded to the Lathe assembly of the *V. atypica* and *E. eligens* bins, the Flye assembly was aligned against the Lathe reference and the best-mapping contigs were retained. More in detail, Flye contigs whose minimap2 primary alignments with MAPQ >= 30 cover at least 80% of their length were retained. CheckM v1.1.2 was hence run on these two sets of retrieved sequences.
