# Supplementary material for: Strainberry: automated strain separation in low-complexity metagenomes using long reads
Source: Nat Commun. 2021 Jul 23;12:4485. doi: 10.1038/s41467-021-24515-9 (PMC8302730; doi:10.1038/s41467-021-24515-9)
Supplement: Supplementary file 3 — Description of Additional Supplementary Files [file 41467_2021_24515_MOESM3_ESM.pdf]

Description of Additional Supplementary Files

File Name: Supplementary Data 1
Description: Assembly evaluation of the Strainberry separated assemblies of the mock and simulated communities. Assemblies obtained with Strainberry using Flye and Canu as input are referred to as ssFlye and ssCanu, respectively. For each dataset, the following measures are displayed: assembly method, reference sequence, number of contigs (#seq), reference size (ref size), assembly size (asm size), NG50, unaligned reference percentage (unaligned ref %), unaligned assembly percentage (unaligned asm %), average nucleotide identity (ANI), duplication ratio (dup ratio), duplicated bases (dup), compressed bases (cmp), single-nucleotide polymorphisms (SNPs), inversions (inv), relocations (reloc).

File Name: Supplementary Data 2
Description: Assembly evaluation of the Strainberry separated assemblies of the NWC2 PacBio and Nanopore datasets. Assemblies obtained with Strainberry using Flye and Canu as input are referred to as ssFlye and ssCanu, respectively. For each dataset, the following measures are displayed: assembly method, reference sequence, number of contigs (#seq), reference size (ref size), assembly size (asm size), NG50, unaligned reference percentage (unaligned ref %), unaligned assembly percentage (unaligned asm %), average nucleotide identity (ANI), duplication ratio (dup ratio), duplicated bases (dup), compressed bases (cmp), single-nucleotide polymorphisms (SNPs), inversions (inv), relocations (reloc). Completeness, contamination, and strain heterogeneity refer to the metrics computed with CheckM on assembled sequences assigned to each reference.

File Name: Supplementary Data 3
Description: Assembly evaluation of the Strainberry separated assemblies of the HSM dataset. The Strainberry assembly obtained using the Lathe reference as input is referred to as ssLathe. QUAST metrics are reported for the Lathe, ssLathe, and (meta)Flye assemblies. The evaluation of the good-quality Lathe bins and their Strainberry separated counterparts is also provided. More precisely, the following details are reported: the assembly method, the bin identifier (bin id), the number of binned sequences (#seq), the cumulative length of binned sequences (bin size), the Kraken2 classification of binned sequences, the number of sequences classified as a specific species/strain (#species sequences), the cumulative length of binned sequences classified as a specific species/strain (assembled size), the fraction of a specific classification in the bin (bin fraction), the completeness, contamination, and strain heterogeneity metrics computed with CheckM, and the average read coverage of the bin (coverage).

File Name: Supplementary Data 4
Description: Assembly evaluation of the Mock3 and Mock9 datasets separated with different strategies. The following measures are displayed: assembly method, reference, number of contigs (#seq), reference size (ref size), assembly size (asm size), NG50, unaligned reference percentage (unaligned ref %), unaligned assembly percentage (unaligned asm %), average nucleotide identity (ANI), duplication ratio (dup ratio), duplicated bases (dup), compressed bases (cmp), single-nucleotide polymorphisms (SNPs), inversions (inv), relocations (reloc). The assembly methods are characterized by the use of either freebayes or Longshot for variant calling and HapCUT2 or Whatshap-polyploid for haplotype phasing and read separation. On the three-strain separation of the Mock9, variant calling was performed either using freebayes alone or providing Longshot calls as input to freebayes (as Longshot does not support polyploid variant calling), while haplotype phasing was performed only with Whatshap-polyploid (as HapCUT2 does not support polyploid phasing).
