# Supplementary material for: Strainberry: automated strain separation in low-complexity metagenomes using long reads
Source: Nat Commun. 2021 Jul 23;12:4485. doi: 10.1038/s41467-021-24515-9 (PMC8302730; doi:10.1038/s41467-021-24515-9)
Supplement: Supplementary file 8 — Reporting Summary [file 41467_2021_24515_MOESM8_ESM.pdf]

nature research

Corresponding author(s): Riccardo Vicedomini

Last updated by author(s): Jun 10, 2021

Reporting Summary

Nature Research wishes to improve the reproducibility of the work that we publish. This form provides structure for consistency and transparency in reporting. For further information on Nature Research policies, see our Editorial Policies and the Editorial Policy Checklist.

Statistics

For all statistical analyses, confirm that the following items are present in the figure legend, table legend, main text, or Methods section.

n/a	Confirmed	
x	☐	The exact sample size (n) for each experimental group/condition, given as a discrete number and unit of measurement
x	☐	A statement on whether measurements were taken from distinct samples or whether the same sample was measured repeatedly
x	☐	The statistical test(s) used AND whether they are one- or two-sided<br>*Only common tests should be described solely by name; describe more complex techniques in the Methods section.*
x	☐	A description of all covariates tested
x	☐	A description of any assumptions or corrections, such as tests of normality and adjustment for multiple comparisons
x	☐	A full description of the statistical parameters including central tendency (e.g. means) or other basic estimates (e.g. regression coefficient) AND variation (e.g. standard deviation) or associated estimates of uncertainty (e.g. confidence intervals)
x	☐	For null hypothesis testing, the test statistic (e.g. F, t, r) with confidence intervals, effect sizes, degrees of freedom and P value noted<br>*Give P values as exact values whenever suitable.*
x	☐	For Bayesian analysis, information on the choice of priors and Markov chain Monte Carlo settings
x	☐	For hierarchical and complex designs, identification of the appropriate level for tests and full reporting of outcomes
x	☐	Estimates of effect sizes (e.g. Cohen's d, Pearson's r), indicating how they were calculated

Our web collection on statistics for biologists contains articles on many of the points above.

Software and code

Policy information about availability of computer code

Data collection

PacBio's software lima v1.11.0 was used for demultiplexing PacBio reads in order to create Mock3 and Mock9 datasets. Badread v0.2.0 was used to generate simulated reads. NCBI sra-tools was used to retrieve raw reads of NWC2 and HSM datasets. NCBI EDirect was used to retrieve all reference sequences used in the study.

Data analysis

The manuscript presents Strainberry, which is open source and publicly available at: https://github.com/rvicedomini/strainberry. Software and scripts used to generate the results and perform the analyses are precisely defined in the snakemake workflow at: https://github.com/rvicedomini/strainberry-analyses. The list of software/packages and their version are also provided hereafter.

Software used either in Strainberry or to perform analyses: Strainberry v1.1, metaFlye v2.7b-b1528 (Mock3 dataset), metaFlye 2.7-b1585 (all other datasets), Canu v2.0, samtools v1.10, minimap2 v2.17, circos v0.69.6, mummer4 v4.0.0beta2, mosdepth v0.2.9, metabat2 v2.15, kraken2 v2.0.9beta, checkm v1.1.2, racon v1.3.2, medaka v0.6.5, marginPolish 1.3.dev-5492204, freebayes v1.3.2, longshot v0.4.1, wtdbg v2.5, whatshap v0.19.dev161 (polyploid-haplotag branch at https://github.com/whatshap/whatshap/tree/polyploid-haplotag), miniconda3 (conda v4.9.2), Python v3.7.9, snakemake v6.0.5

Python packages used either in Strainberry or to perform analyses: pysam v0.16.0.1, biopython v1.78, numpy v1.19.1, scipy v1.2.1, pandas v1.0.3, seaborn v0.10.0, matplotlib v3.1.3, networkx v2.5, pygraphviz v1.3, pyvcf v0.6.8

For manuscripts utilizing custom algorithms or software that are central to the research but not yet described in published literature, software must be made available to editors and reviewers. We strongly encourage code deposition in a community repository (e.g. GitHub). See the Nature Research guidelines for submitting code & software for further information.

Data

Policy information about availability of data

All manuscripts must include a data availability statement. This statement should provide the following information, where applicable:

- Accession codes, unique identifiers, or web links for publicly available datasets
- A list of figures that have associated raw data
- A description of any restrictions on data availability

All described datasets are publicly available. Links and accession codes are provided in this section. PacBio sequencing data used to create the Mock3 and Mock9 datasets is available at https://github.com/PacificBiosciences/DevNet/wiki/Microbial-Multiplexing:-PacBio-Sequel-System,-Chemistry---v3.0,-Analysis---SMRT-Link-v6.0.0. NCBI RefSeq accession codes of the reference genomes used for the assembly evaluation and the generation of the simulated mock communities are listed in the Supplementary Information (Supplementary Tables 1 and 2). NWC2 reads are available at the NCBI BioSample SAMN09580370 under the SRA accession codes SRX4451758 (Nanopore) and SRX4451757 (PacBio). Reference sequences used for the assembly evaluation of NWC2 are accessible under the NCBI BioSample accession codes SAMN09476686 (S. thermophilus), SAMN09476687 (L. delbrueckii), SAMN09476688 (L. helveticus strain NWC_2_3), and SAMN09476689 (L. helveticus strain NWC_2_4). HSM reads and the corresponding metagenome assembly reference are available at the NCBI BioProject PRJNA508395 under the SRA accession code SRX5235113 and the GenBank accession code GCA_011075405.1, respectively. All assemblies generated with metaFlye, Canu, and Strainberry are available at https://doi.org/10.5281/zenodo.4721347.

Field-specific reporting

Please select the one below that is the best fit for your research. If you are not sure, read the appropriate sections before making your selection.

☒ Life sciences ☐ Behavioural & social sciences ☐ Ecological, evolutionary & environmental sciences

For a reference copy of the document with all sections, see nature.com/documents/nr-reporting-summary-flat.pdf

Life sciences study design

All studies must disclose on these points even when the disclosure is negative.

Sample size	Does not apply. This study does not include statistical analysis of any hypothesis
Data exclusions	Reads below a certain length threshold (10 Kbp for ONT, 5 Kbp for PacBio) were discarded from the NWC2 dataset. This was done in order to evaluate generated assemblies using the same raw long-read data used to generate the reference sequences in the study of Somerville et al. (2019), from which the data was collected and evaluated.
Replication	Datasets, software, analysis scripts, and generated assemblies are all publicly available (see "Data availability" and "Code availability" sections). Strainberry and the analysis workflow make use of specific software versions in order to ensure the best possible reproducibility of the results. Nevertheless, due to an unavoidable non-deterministic behavior of assemby tools (metaFlye, Canu, wtdbg2), it is not possible to reproduce exactly the results presented in the manuscript. The analysis workflow was run multiple times to assess nearly identical results.
Randomization	Does not apply. This study introduces a method and does not include biological hypotheses analysis
Blinding	Does not apply. This study introduces a method and does not include biological hypotheses analysis

Reporting for specific materials, systems and methods

We require information from authors about some types of materials, experimental systems and methods used in many studies. Here, indicate whether each material, system or method listed is relevant to your study. If you are not sure if a list item applies to your research, read the appropriate section before selecting a response.

Materials & experimental systems

n/a	Involved in the study
☒	☐ Antibodies
☒	☐ Eukaryotic cell lines
☒	☐ Palaeontology and archaeology
☒	☐ Animals and other organisms
☒	☐ Human research participants
☒	☐ Clinical data
☒	☐ Dual use research of concern

Methods

n/a	Involved in the study
☒	☐ ChIP-seq
☒	☐ Flow cytometry
☒	☐ MRI-based neuroimaging
